# RNA duplexes with abasic substitutions are potent and allele-selective inhibitors of huntingtin and ataxin-3 expression

**DOI:** 10.1093/nar/gkt594

**Published:** 2013-07-24

**Authors:** Jing Liu, Hannah Pendergraff, K. Jayaprakash Narayanannair, Jeremy G. Lackey, Satya Kuchimanchi, Kallanthottathil G. Rajeev, Muthiah Manoharan, Jiaxin Hu, David R. Corey

**Affiliations:** ^1^Departments of Pharmacology and Biochemistry, UT Southwestern Medical Center, 6001 Forest Park Road, Dallas, TX 75390-9041, USA, ^2^Department of Chemistry and Institute for Life Sciences, University of Southampton, Highfield, Southampton, SO17 1BJ, UK and ^3^Alnylam Pharmaceuticals, 300 Third St., Cambridge, MA 02142, USA

## Abstract

Abasic substitutions within DNA or RNA are tools for evaluating the impact of absent nucleobases. Because of the importance of abasic sites in genetic damage, most research has involved DNA. Little information is available on the impact of abasic substitutions within RNA or on RNA interference (RNAi). Here, we examine the effect of abasic substitutions on RNAi and allele-selective gene silencing. Huntington's disease (HD) and Machado Joseph Disease (MJD) are severe neurological disorders that currently have no cure. HD and MJD are caused by an expansion of CAG repeats within one mRNA allele encoding huntingtin (HTT) and ataxin-3 (ATX-3) proteins. Agents that silence mutant HTT or ATX-3 expression would remove the cause of HD or MJD and provide an option for therapeutic development. We describe flexible syntheses for abasic substitutions and show that abasic RNA duplexes allele-selectively inhibit both mutant HTT and mutant ATX-3. Inhibition involves the RNAi protein argonaute 2, even though the abasic substitution disrupts the catalytic cleavage of RNA target by argonaute 2. Several different abasic duplexes achieve potent and selective inhibition, providing a broad platform for subsequent development. These findings introduce abasic substitutions as a tool for tailoring RNA duplexes for gene silencing.

## INTRODUCTION

Many modifications exist to alter the properties of RNA or DNA oligonucleotides to make them better suited to laboratory applications or therapeutic development (1). One modification that has received relatively little attention results from incorporation of a residue without a base. Abasic sites occur spontaneously in cellular DNA at a frequency of approximately 1 in 300 000 bases per genome per day (2). Because abasic sites can lead to genomic damage, synthetic abasic monomers are often used to construct model DNA strands for study of the cellular machinery for DNA damage repair.

Less attention has been paid to abasic site-containing RNA (abasic RNA), even though the modification modulates physicochemical properties and biological function. RNA that contains abasic sites is more stable than abasic site-containing DNA (3,4). Abasic RNA can interact with enzymes, including HIV reverse transcriptase (4,5), APE1 endonuclease (6,7) and mutated *Thermococcus* DNA polymerase (8). Abasic RNA has been used to probe RNA structure (9) and can be compatible with RNA interference (RNAi) (10). These studies provide a starting point for using abasic RNA as a tool for research and discovery.

For treatment of certain diseases like Huntington’s disease (HD) and Machado Joseph Disease [MJD also known as spinocerebellar ataxia 3 (SCA3)] that are caused by expansion of CAG repeats within one allele of the mRNA, allele selectivity is desired for gene-targeting agents like small interfering RNAs (siRNAs). We previously focused on using mismatched RNA to modulate RNAi activity to achieve allele selectivity (11–15). RNA duplexes with centrally located mismatches disrupt argonaute-2 (AGO2)-mediated cleavage of target mRNA (16). We, and others, have observed that mismatched RNA duplexes enable discrimination between the wild-type and mutant alleles of *huntingtin* (*HTT*), the gene responsible for HD (11,13–15,17). Our data suggest that by disrupting the potential for cleavage, mismatched duplexes are better able to discriminate between the wild-type and mutant *HTT* alleles.

HD is an incurable neurological disorder (18,19) caused by an expansion within a CAG trinucleotide repeat near the 5′ translation start site in the *HTT* mRNA. Wild-type *HTT* contains fewer than 26 CAG repeats. Patients with mutant *HTT* containing more than 37 repeats may show disease symptoms. Afflicted patients have *HTT* with average of 45 repeats. Agents that selectively inhibit the expression of mutant HTT protein would be ideal agents for treating HD. MJD is caused by an expansion within the gene encoding ataxin-3 protein (ATX-3) (20,21). Because MJD, HD and other diseases share a common molecular defect, it is possible that a single anti-CAG agent may be able to treat multiple pathologies.

Considerable progress has been made in developing antisense oligonucleotides (22,23) and duplex RNAs (24–32) as inhibitors of HTT or MJD expression. Both modalities can repress gene expression and offer a near-term option for clinical development. The challenges of identifying agents capable of potent and selective action in the central nervous system, combined with the urgent needs of HD patients, make identification of improved agents a priority.

Like mismatched bases, introduction of abasic sites will remove the potential for normal base-pairing. Unlike mismatched bases, abasic sites eliminate stacking interactions and any potential for formation of suboptimal or wobble base pairs. Thus, abasic substitutions may offer a distinct and unexplored alternative to mismatched bases as a strategy to modulate the function of siRNAs and other nucleic acid-based therapeutic agents. Specifically, for anti-CAG duplexes designed to inhibit expression of HTT or ATX-3, abasic substitutions would widen the pool of compounds available for optimizing properties for *in vivo* inhibition. The use of abasic substitutions to promote allele selectivity has not been examined previously.

In this study, we demonstrate that substitution of one or more sites in the central region of the antisense strand of an siRNA duplex is compatible with the RNAi machinery. With siRNAs containing these modified residues, we observed recruitment of AGO2 but no cleavage of mRNA targets. This approach resulted in potent and allele-selective inhibition of expression for mutant HTT and ATX-3 proteins in cell-based assays. Abasic duplexes provide an alternative for modulating the mechanism of RNAi machinery to tailor the activity of small duplex RNAs to fit the needs of basic and biomedical research.

## MATERIALS AND METHODS

### Cell culture and transfection

Chemically modified RNA oligonucleotides were synthesized at Alnylam Pharmaceuticals, and synthetic protocols are provided as Supplementary Information. The melting temperature of siRNAs was measured using a CARY Varian spectrophotometer. Patient-derived fibroblast cell lines GM04281 and GM06151 were obtained from the Coriell Institute. The fibroblasts were maintained at 37°C and 5% CO_2_ in Minimal Essential Media Eagle (MEM, Sigma) supplemented with 10% heat-inactivated fetal bovine serum (Sigma) and 0.5% MEM nonessential amino acids (Sigma). siRNAs were transfected into cells using RNAiMAX (Invitrogen) using manufacturer’s protocol (33). Cells were typically harvested 3 days after transfection for analysis of mRNA levels by quantitative polymerase chain reaction (qPCR) or 4 days after transfection for analysis of protein levels.

### Western blot and qPCR analysis

Mutant and wild-type HTT proteins were separated on 5% acrylamide gels (33,34). Pre-cast 7.5% acrylamide gels (Bio-Rad) were used to separate the ataxin-3 isoforms. The following primary antibodies were used: anti-HTT (MAB2166, Chemicon Millipore), anti-ataxin-3 (MAB5360, Chemicon Millipore) and anti-β-actin (Sigma). Protein bands were quantified using ImageJ software. The percentage of inhibition was calculated as a relative value to a control sample. Dose–response curves were generated using GraphPad Prism 4 and the equation: y = 100 [1−x^m^/(n^m ^+ x^m^)], where y is percentage of inhibition and x is the siRNA concentration, m and n are fitting parameters, where n is the IC_50_ value. All experiments were repeated at least three times, and the error bars indicate standard deviation.

qPCR was performed on a 7500 real-time PCR system (Applied Biosystems) using iTaq SYBR Green Supermix (Bio-Rad). Data were normalized relative to levels of 18S rRNA. The following primers were used for amplification: HTT forward: 5′-CGACAGCGAGTCAGTGAATG-3′; HTT reverse: 5′-ACCACTCTGGCTTCACAAGG-3′; 18S rRNA primers were obtained from Applied Biosystems.

### *In vitro* cleavage assay

The RNA transcript containing *HTT* exon1 with 17 CAG repeats was synthesized by *in vitro* transcription from cloned fragments of the *HTT* gene and gel purified (34). The RNA substrate was labeled with ^32^P-ATP after dephosphorylation. We pre-incubated 250 nM of 5′-phosphorylated siRNA antisense strand and purified recombinant human AGO2 protein (generously provided by Dr. Qinghua Liu) at room temperature in 2 µl 10× reaction buffer (0.5 M Tris, pH 7.4, 20 mM MgCl_2_, 5 mM DTT, 2.5 mM ATP, 1 M KCl, 0.5 M NaCl) for 1.5 h. The ^32^P-labeled RNA substrate was added, and the reaction was incubated for 1.5 h at 37°C. After phenol/chloroform extraction, the RNA was precipitated with 2% LiClO_4_ acetone and separated on a 14% acrylamide/7 M urea gel.

### RNA immunoprecipitation

HTT fibroblast cells (1.4 × 10^6^ cells) were seeded in 150-cm^2^ dishes (35). Cells were transfected with RNAs using RNAiMAX the next day. Cells were harvested 72 h later and lysed in buffer (20 mM Tris–HCl, pH 7.4, 150 mM NaCl, 2 mM MgCl_2_, 0.5% NP-40, 0.5 mM DTT) containing protease inhibitor (EDTA-free, Roche) and RNase inhibitor (Promega, 50 U/ml final) in a volume about 3 times that of the cell pellet. After thorough mixing, samples were placed on ice for 10 min. After centrifugation, the supernatants were isolated and stored at −80°C.

Protein A/G agarose Plus (60 µl) was incubated with 5 µl of anti-AGO2 antibody (015-22031, Wako) in phosphate buffered saline (pH 7.4) at 4°C with gentle agitation for 2 h (35). After two washes with phosphate buffered saline and one wash with lysis buffer, beads were incubated with cell lysate for 2 h at 4°C. The beads were extensively washed with lysis buffer three times. RNA was eluted from the beads with elution buffer (1% SDS, 0.1 M NaHCO_3_ and RNase inhibitor). After proteinase K treatment, RNA extraction and precipitation, samples were treated with recombinant DNase I. After reverse transcription, the *HTT* mRNA levels were quantified by qPCR.

## RESULTS

### Synthesis of siRNAs containing abasic moieties

The abasic moieties were introduced into the oligonucleotides using phosphoramidites of the corresponding abasic residues compatible with solid-phase oligonucleotide synthesis and deprotection conditions. The oligonucleotides were synthesized on a MerMade synthesizer; coupling time and reagent conditions for the abasic monomers were similar to standard deoxy- and ribo-3′-phosphoramidites.

**Figure 1.**
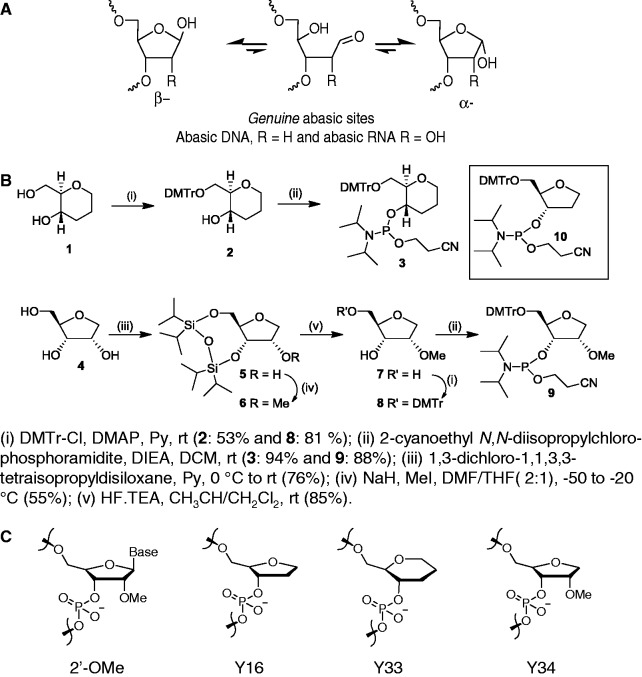
(**A**) Structures of genuine abasic sites. (**B**) Synthesis of abasic phosphoramidite monomers. (**C**) Structures of synthetic abasic mimics and 2′-OMe RNA used in this study. 2′-OMe: 2′-O-methyl RNA; Y16: (2*R*,3*S*)-2 -(hydroxymethyl)tetrahydrofuran-3-phosphate; Y33: (2*R*,3*S*)-2 -(hydroxymethyl)tetrahydro-2H-pyran-3-phosphate; Y34: (2*R*,3*S*,4*S*)-2-hydroxymethyl-4-methoxytetrahydrofuaran-3-phosphate.

We evaluated three variants of the abasic moieties derived from furanose and pyranose sugars. For synthetic convenience and to avoid having anomeric mixtures, the anomeric hydroxyl group on genuine DNA and RNA abasic residues (Figure 1A) (4) was eliminated from the design (Figure 1B). The three variants of the abasic moieties evaluated are: (i) (2*R*,3*S*)-2 -(hydroxymethyl)tetrahydrofuran-3-ol (pentafuranose abasic with 2-deoxy, Y16), (ii) (2*R*,3*S*,4*S*)-2-hydroxymethyl-4-methoxytetrahydrofuaran-3-ol (pentafuranose abasic with 2-OMe, Y34) and (iii) (2*R*,3*S*)-2 -(hydroxymethyl)tetrahydro-2H-pyran-3-ol (hexapyranose abasic moiety, Y33) (Figure 1C). In addition to abasic moieties, 2′-*O*-methylribosugar modification (2′-OMe, Figure 1C) was introduced into selected oligonucleotides at desired positions.

Therapeutics based on synthetic RNA oligonucleotides require chemical modifications to improve biodistribution and tissue half-lives (1). The introduction of 2′-*O*-methylribosugar modification (2′-OMe, Figure 1C) improves resistance of modified RNAs to nucleases and reduces the likelihood of provoking off-target effects due to the interferon response. Some of the duplexes tested here contained 2′-OMe RNA.

To introduce abasic moieties Y33 and Y34 into the guide strand, the phosphoramidite monomers 3 and 9 were synthesized from (2*R*,3*S*)-2 -(hydroxymethyl)tetrahydro-2H-pyran-3-ol (1) and (2 *R*,3*S*,4*S*)-2-hydroxymethyl-4-methoxytetrahydrofuaran-3-ol (4), respectively (Figure 1B). Commercially available phosphoramidite 10 was used to introduce abasic moiety Y16 to oligonucleotides. We synthesized duplexes containing one or more centrally located abasic substitutions on the guide strand, extensive 2′-OMe ribosugar modifications on the passenger strand and single phosphorothioate substitutions near the 3′ termini of both strands. Annealing of the oligonucleotides containing abasic moieties with equimolar amount of the corresponding complementary strands afforded the abasic siRNA shown in Tables 1 and 2.

**Table 1.** Inhibition of HTT expression by duplex RNAs with single abasic substitution

RNA	Sequence	Mismatch Position	Modification	T_m_ (°C)	Mut IC_50_ (nM)	WT IC_50_ (nM)	Selectivity
CMOD24	GCUGCUGcUGCUGCUGCUGdT_s_dT	–	2′-OMe	87.9	–	–	–
	dT_s_dTcGAcGAcGAcGAcGAcGAc						
CMOD25	GCUGCUGcU**A**CUGCUGCUGdT_s_dT	G10	2′-OMe	84.3	–	–	–
	dT_s_dTcGAcGAcGA**u**GAcGAcGAc						
CMOD26	GCUGCUGcUG**A**UGCUGCUGdT_s_dT	G11	2′-OMe	85.0	–	–	–
	dT_s_dTcGAcGAcGAc**u**AcGAcGAc						
AB1	UGCUGCUGc**Y****_34_**GCUGCUGCUdT_s_dT	U10	Y34	79.8	–	–	–
	dT_s_dTAcGAcGAcGAcGAcGAcGA						
AB2	UGCUGCUGc**Y****_33_**GCUGCUGCUdT_s_dT	U10	Y33	78.4	–	–	–
	dT_s_dTAcGAcGAcGAcGAcGAcGA						
AB3	GCUGCUGc**Y****_34_**GCUGCUGCUGdT_s_dT	G9	Y34	79.9	3.0 ± 0.4	75 ± 30	25
	dT_s_dTcGAcGAcGAcGAcGAcGAc						
AB4	GCUGCUGcU**Y****_34_**CUGCUGCUGdT_s_dT	G10	Y34	73.9	3.7 ± 1.3	>100	>27
	dT_s_dTcGAcGAcGA**u**GAcGAcGAc						
AB5	GCUGCUGcUG**Y****_34_**UGCUGCUGdTsdT	G11	Y34	74.3	2.5 ± 0.5	>100	>40
	dT_s_dTcGAcGAcGAc**u**AcGAcGAc						
AB6	GCUGCUGc**Y****_33_**GCUGCUGCUGdT_s_dT	G9	Y33	79.8	2.8 ± 0.4	>100	>36
	dT_s_dTcGAcGAcGAcGAcGAcGAc						
AB7	GCUGCUGcU**Y****_33_**CUGCUGCUGdT_s_dT	G10	Y33	75.3	5.8 ± 4.0	>100	>17
	dT_s_dTcGAcGAcGA**u**GAcGAcGAc						
AB8	GCUGCUGcUG**Y****_33_**UGCUGCUGdT_s_dT	G11	Y33	75.8	3.3 ± 0.6	>100	>30
	dT_s_dTcGAcGAcGAc**u**AcGAcGAc						

The antisense strand of the siRNA is shown from 5′ to 3′ and the sense strand is from 3′ to 5′.

Mismatched bases and abasic sites are in bold. Lower case indicates 2′-OMe nucleotide modification. siRNAs were tested in HD patient fibroblasts GM04281 (mutant allele/69 CAG, wild type/17 CAG repeats). The letter “G” or “U” before the number under “Mismatch position” refers to the 3′ terminal base of the antisense strand.

**Table 2.** Inhibition of HTT expression by duplex RNAs with multiple abasic substitutions

RNA	Sequence	Mismatch Position	Modification	T_m_ (°C)	Mut IC_50_ (nM)	WT IC_50_ (nM)	Selectivity
AB9	GCUGCUGc**Y****_16_****Y****_16_**CUGCUGCUGdT_s_dT	G9,10	Y16	68.6	6.1 ± 1.4	>100	16
	dT_s_dTCGACGACG**AA**GACGACGAC						
AB10	GCUGCUGc**Y****_16_****Y****_16_****Y****_16_**UGCUGCUGdT_s_dT	G9,10,11	Y16	63.8	29 ± 8.5	>100	3.5
	dT_s_dTCGACGACG**AAA**ACGACGAC						
AB11	GCUGCUGc**Y****_16_****Y****_16_****Y****_16_****Y****_16_**GCUGCUGdT_s_dT	G9,10,11,12	Y16	62.8	49 ± 6.5	>100	2
	dT_s_dTCGACGACG**AAAA**CGACGAC						
AB12	GCUGCUGc**Y****_33_****Y****_33_**CUGCUGCUGdT_s_dT	G9,10	Y33	68.7	3.4 ± 1.2	>100	29
	dT_s_dTCGACGACG**AA**GACGACGAC						
AB13	GCUGCUGc**Y****_33_****Y****_33_****Y****_33_**UGCUGCUGdT_s_dT	G9,10,11	Y33	64.3	15 ± 4.2	>100	6.6
	dT_s_dTCGACGACG**AAA**ACGACGAC						
AB14	GCUGCUGc**Y****_33_****Y****_33_****Y****_33_****Y****_33_**GCUGCUGdT_s_dT	G9,10,11,12	Y33	62.2	34 ± 11	>100	2.9
	dT_s_dTCGACGACG**AAAA**CGACGAC						
AB15	GCUGCUG**Y****_33_****Y****_33_**G**Y****_33_**UGCUGCUGdT_s_dT	G8,9,11	Y33	59.5	43 ± 7.4	>100	2.3
	dT_s_dTCGACGAC**AA**C**A**ACGACGAC						
AB16	GCUGCUG**Y****_33_****Y****_33_****Y****_33_****Y****_33_**UGCUGCUGdT_s_dT	G8,9,10,11	Y33	59.1	>100	>100	–
	dT_s_dTCGACGAC**AAAA**ACGACGAC						
AB17	GCUGCUGCUG**Y****_33_**UGCU**Y****_33_**CUGdT_s_dT	G11,16	Y33	62.7	47 ± 15	>100	2
	dT_s_dTCGACGACGAC**A**ACGA**A**GAC						
AB18^a^	UGCUGCu**Y****_33_****Y****_33_**u**Y****_33_**CUGCUGCUGCUdT_s_dT	U,8,9,11	Y33	68.1	5.2 ± 2.2	>100	19
	dT_s_dTACGACGA**AA**A**A**GACGACGACGA						
AB19^a^	UGCUGCu**Y****_33_****Y****_33_****Y****_33_**GCUGCUGCUGCUdT_s_dT	U,8,9,10	Y33	72.8	3.8 ± 1.4	>100	26
	dT_s_dTACGACGA**AAA**CGACGACGACGA						
AB20^a^	UGCUGCu**Y****_16_****Y****_16_**u**Y****_16_**CUGCUGCUGCUdT_s_dT	U,8,9,11	Y16	68.0	20 ± 2.4	>100	5
	dT_s_dTACGACGA**AA**A**A**GACGACGACGA						
AB21^a^	UGCUGCu**Y****_16_****Y****_16_****Y****_16_**GCUGCUGCUGCUdT_s_dT	U,8,9,10	Y16	73.3	11 ± 1.5	>100	9
	dT_s_dTACGACGA**AAA**CGACGACGACGA						

^a^Represents 22-mer duplex, others are 19 mers.

The antisense strand of the siRNA is shown from 5′ to 3′ and the sense strand is from 3′ to 5′.

Mismatched bases and abasic sites are in bold. siRNAs were tested in HD patient fibroblasts GM04281 (mutant allele/69 CAG, wild-type/17 CAG repeats). The letter “G” or “U” before the number under “Mismatch position” refers to the 3′ terminal base of the antisense strand.

### Allele-selective inhibition by chemically modified duplexes containing base-mismatches

In our previous studies, our allele-selective siRNA duplexes with central mismatches were not chemically modified (11,14). Although unmodified duplex RNA is an excellent tool for investigations in cell culture, it is likely that chemical modifications will be necessary for testing in animal models or for clinical development (1). Such modifications stabilize the duplex against digestion by nucleases and may improve biodistribution and pharmacokinetics.

We set out to test whether the modifications necessary for *in vivo* applications would compromise allele selectivity and be a plausible background for subsequent introduction of abasic RNA. We synthesized anti-CAG duplexes containing the 2′-OMe sugar modification and evaluated silencing of the mutant and wild-type alleles of *HTT*. The duplexes also contained single phosphorothioate substitutions near the 3′ termini of both passenger and guide strands for added exonuclease stability and one or more mismatched bases between the guide strand and the targeted allele.

**Figure 2.**
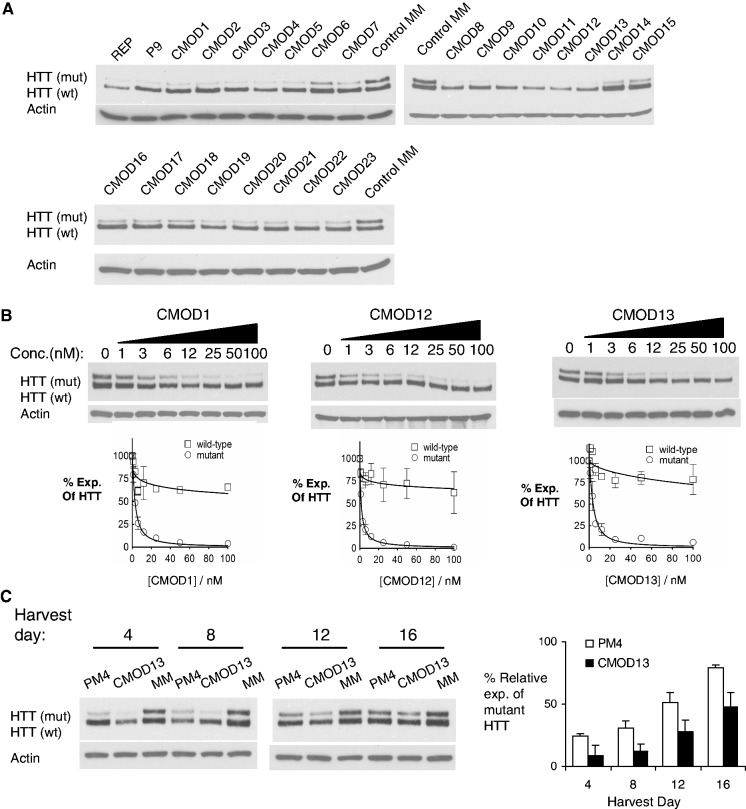
Effects of chemically modified duplex RNAs with mismatched bases on HTT expression. Duplex RNAs were transfected into HD patient-derived cells (GM04281, CAG 69/17) (**A**) Representative western blot images of HTT expression after transfection of duplex RNAs (Table 3) at 25 nM. (**B**) Western blot images and dose curves of duplex RNAs CMOD1, CMOD12 and CMOD13. Dose curves are averaged data from three independent experiments. (**C**) Effects of chemically modified RNA CMOD13 and analogous unmodified RNA duplex PM4 on HTT expression. Duplex RNAs were added at 25 nM and harvested at indicated days after transfection. The bar graph shows quantification of mutant HTT protein levels relative to treatment with a control duplex MM4 that contained mismatches within the seed sequence.

We transfected the anti-CAG duplexes into GM04281 HD patient-derived fibroblast cells (mutant allele 69 CAG repeats/wild-type allele 17 repeats) with cationic lipid and monitored inhibition of HTT protein expression using gel electrophoresis to separate the mutant (upper band) and wild-type (lower band) proteins. Several chemically modified duplexes with one or two mismatches showed allele specificity in inhibition of gene expression (Table 3, Figure 2A). Duplex CMOD1 (CMOD = Chemically MODified) had an IC_50_ value of 2.8 nM and an allele selectivity of >36 fold (Figure 2B). These values are similar to those achieved with unmodified RNA duplexes (11,14), showing that chemical modifications were compatible with potent and allele-selective gene silencing.

**Table 3.** Inhibition of HTT expression by chemically modified mismatch-containing duplex RNAs

RNA	Sequence	Mismatch Position^a^	T_m_ (°C)	Mut IC_50_ (nM)	WT IC_50_ (nM)	Selectivity
CMOD1	GCUGCUGc**A**GCUGCUGCUGdT_s_dT	G9	89.2	2.8 ± 0.2	>100	>36
	dT_s_dTcGAcGAcG**u**cGAcGAcGAc					
CMOD2	GCUGCUGc**AA**CUGCUGCUGdT_s_dT	G9,10	85.5	2.8 ± 0.3	>100	>36
	dT_s_dTcGAcGAcG**uu**GAcGAcGAc					
CMOD3	GCUGCUGc**A**GCUGCUGCUGdT_s_dT	G9^b^	81.1	3.3 ± 0.3	>100	>30
	dT_s_dTcGAcGAcGAcGAcGAcGAc					
CMOD4	GCUGCUGCu**A**CUGCUGCUGdT_s_dT	G10^b^	79.8	6.8 ± 0.7	>100	>15
	dT_s_dTcGAcGAcGAcGAcGAcGAc					
CMOD5	GCUGCUGCUG**A**UGCUGCUGdT_s_dT	G11^b^	78.4	4.9 ± 0.6	>100	>20
	dT_s_dTcGAcGAcGAcGAcGAcGAc					
CMOD6	CUGCUGCUG**A**UGCUGCUGCdT_s_dT	C10*	78.2	7.1 ± 1.3	>100	>14
	dT_s_dTGAcGAcGAcGAcGAcGAcG					
CMOD7	UGCUGCUGc**A**GCUGCUGCUdT_s_dT	U10^b^	80.2	5.1 ± 1.2	>100	>20
	dT_s_dTAcGAcGAcGAcGAcGAcGA					
CMOD8	GCUGCUG**A**u**A**CUGCUGCUGdT_s_dT	G8,10	83.2	–	–	–
	dT_s_dTCGAcGAc**u**A**u**GACGAcGAc					
CMOD9	GCUGCUG**A**UG**A**UGCUGCUGdT_s_dT	G8,11	82.8	–	–	–
	dT_s_dTCGAcGAc**u**Ac**u**ACGAcGAc					
CMOD10	GCUGCUG**AAA**CUGCUGCUGdT_s_dT	G8,9,10	82.1	5.1 ± 0.6	84 ± 17	16
	dT_s_dTCGAcGAc**uuu**GACGAcGAc					
CMOD11	GCUGCUG**AA**G**A**UGCUGCUGdT_s_dT	G8,9,11	82.9	3.3 ± 0.7	67 ± 12	20
	dT_s_dTCGAcGAc**uu**c**u**ACGAcGAc					
CMOD12	GCUGCUGc**AAA**UGCUGCUGdT_s_dT	G9,10,11	82.2	1.4 ± 0.5	>100	>71
	dT_s_dTCGAcGAcG**uuu**ACGAcGAc					
CMOD13	GCUGCUG**AAAA**UGCUGCUGdT_s_dT	G8,9,10,11	78.6	3.6 ± 1.3	>100	>28
	dT_s_dTCGAcGAc**uuuu**ACGAcGAc					
CMOD14	CUGCUGc**AAA**UGCUGCUGCdT_s_dT	C8,9,10	82.0	20 ± 2.7	>100	>5
	dT_s_dTGAcGAcG**uuu**AcGAcGAcG					
CMOD15	CUGCUGc**AA**c**A**GCUGCUGCdT_s_dT	C8,9,11	85.7	–	–	–
	dT_s_dTGAcGAcG**uu**G**u**cGAcGAcG					
CMOD16	CUGCUGCu**AAA**GCUGCUGCdT_s_dT	C9,10,11	82.7	–	–	–
	dT_s_dTGAcGAcGA**uuu**cGAcGAcG					
CMOD17	CUGCUGc**AAAA**GCUGCUGCdT_s_dT	C8,9,10,11	82.7	19 ± 4.2	>100	>5
	dT_s_dTGAcGAcG**uuuu**cGAcGAcG					
CMOD18	UGCUGCu**AA**UGCUGCUGCUdTsdT	U8,9	81.6	–	–	–
	dT_s_dTACGAcGA**uu**AcGACGAcGA					
CMOD19	UGCUGCUG**A**u**A**CUGCUGCUdT_s_dT	U9,11	81.8	–	–	–
	dT_s_dTACGAcGAc**u**A**u**GACGAcGA					
CMOD20	UGCUGCu**AAA**GCUGCUGCUdT_s_dT	U8,9,10	81.9	2.8 ± 0.5	>100	>36
	dT_s_dTACGAcGA**uuu**cGACGAcGA					
CMOD21	UGCUGCu**AA**u**A**CUGCUGCUdT_s_dT	U8,9,11	79.7	8.6 ± 4.1	>100	>12
	dT_s_dTACGAcGA**uu**A**u**GACGAcGA					
CMOD22	UGCUGCUG**AAA**CUGCUGCUdT_s_dT	U9,10,11	83.5	2.7 ± 1.1	>100	>37
	dT_s_dTACGAcGAc**uuu**GACGAcGA					
CMOD23	UGCUGCu**AAAA**CUGCUGCUdT_s_dT	U8,9,10,11	79.3	7.9 ± 3.2	>100	>13
	dT_s_dTACGAcGA**uuuu**GACGAcGA					

^a^G9 represents that the antisense sequence starts with base G and the mismatched base is at position 9.

^b^Represents the duplex contains mismatched base only in its antisense strand. siRNAs were tested in HD patient fibroblasts GM04281 (mutant allele/69 CAG, wild-type/17 CAG repeats). Selectivity is calculated by comparing the IC_50_ for inhibiting wild-type HTT versus the IC_50_ for the mutant allele.

The antisense strand of the siRNA is shown from 5′ to 3′ and the sense strand is from 3′ to 5′.

Lowercase indicates 2′-OMe nucleotide modification. Mismatched bases are in bold. The letter “G” or “U” before the number under “Mismatch position” refers to the 3′ terminal base of the antisense strand.

We then designed a series of duplexes (Table 3) that contained multiple mismatches to minimize complementarity to other sequences within the genome and identified more siRNA candidates (Table 3, Figure 2B). Several of these compounds were assayed at different concentrations. Duplex CMOD12 with three central mismatches was the most potent and the most selective, with an IC_50_ value of 1.4 nM and a selectivity of >71 fold.

Slight manipulations of mismatch position resulted in differences in specificity and efficacy profiles. For example, CMOD13 (four mismatches) possessed an IC_50_ value of 3.6 nM and a selectivity of >28. These data indicate that slight manipulation of mismatch position not only can increase the discrimination of duplex RNAs for targets within the transcriptome, but can also lead to superior allele selectivity. Potent and allele-selective inhibition was not just a property of one or a few duplexes, it appears to be achievable by many different mismatched duplex designs.

We also examined inhibition as a function of time after transfection of CMOD13 and observed at least partial inhibition of mutant allele expression after 16 days (Figure 2C). This long-lasting inhibition was achieved even though the RNA was added once and GM04281 cells in culture typically double in population every 3 days. Inhibition at later time points was superior to that produced by PM4, an unmodified RNA duplex. These data demonstrate that chemical modifications confer prolonged biological effects relative to the native duplex RNA and show superior properties even in the relatively benign environment of cell culture assays.

### Allele-selective inhibition by abasic duplexes

**Figure 3.**
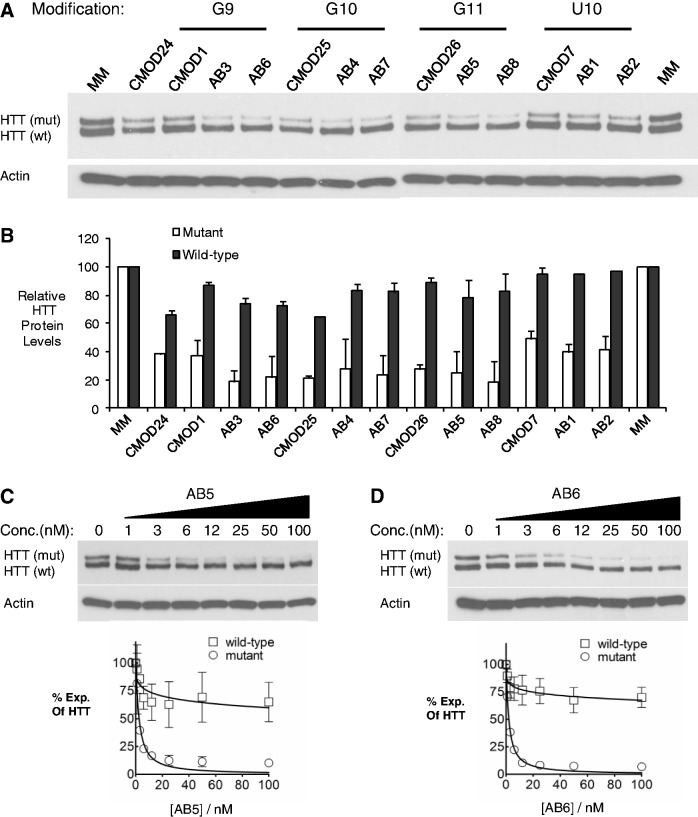
Effects of duplex RNAs with abasic modifications on expression of HTT. Duplexes were transfected into HD patient-derived fibroblast cells (GM04281, CAG 69/17). (**A**) Representative western blot images and (**B**) averaged protein levels of HTT expression after transfection of duplex RNAs at 25 nM. Western blot images and dose curves of (**C**) duplex RNA AB5 and (**D**) duplex RNA AB6. Dose curves are averaged data from three independent experiments. MM:noncomplementary duplex RNA.

Several abasic siRNAs were potent and allele-selective inhibitors (Figure 3A and B). For example, AB5 with one Y34 substitution at position 11 of the guide strand produced >40-fold allele-selective inhibition with an IC_50_ value of 2.5 nM (Figure 3C). AB6, a Y33-substituted duplex, was similarly effective with an IC_50_ value of 2.8 nM and selectivity of >36 fold (Figure 3D). These data demonstrate that abasic substitutions are compatible with efficient RNAi and are a general platform for achieving allele-selective inhibition. Despite the structural differences between an abasic residue and a nucleotide mismatch, the potencies and allele selectivities of abasic duplexes are comparable with analogous mismatch-containing duplexes.

**Figure 4.**
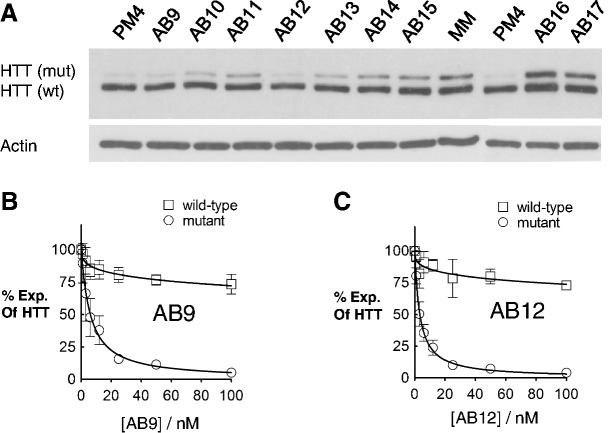
Effects of siRNAs with multiple abasic modifications on expression of HTT. Duplex RNAs were tested in HD patient-derived fibroblast cells (GM04281, CAG 69/17). (**A**) Representative western blot images of HTT expression after treating with 25 nM of siRNAs. Averaged dose curves showing HTT expression after treating with increased concentrations of duplex RNAs (**B**) AB9 and (**C**) AB12.

We also examined duplexes containing multiple abasic substitutions and identified additional allele-selective inhibitors of HTT expression (Figure 4A and Supplementary Figure S1A, Table 2). The most effective duplexes were AB9, a duplex with two Y16 substitutions, an IC_50_ value of 6.1 nM and a selectivity of >16 fold (Figure 4B and Supplementary Figure S1B), and AB12, a duplex with two Y33 substitutions, an IC_50_ value of 3.4 nM and a selectivity of >29 fold (Figure 4C and Supplementary Figure S1B).

### Inhibition of HTT expression by 22 base-pair duplexes

Introducing multiple abasic substitutions had a greater impact on inhibition than introducing multiple mismatches. We found that duplexes with three or four abasic substitutions were less potent inhibitors of HTT expression than their singly or doubly substituted counterparts. By contrast, duplexes with three or four mismatches were as potent as duplexes with one or two mismatches.

**Figure 5.**
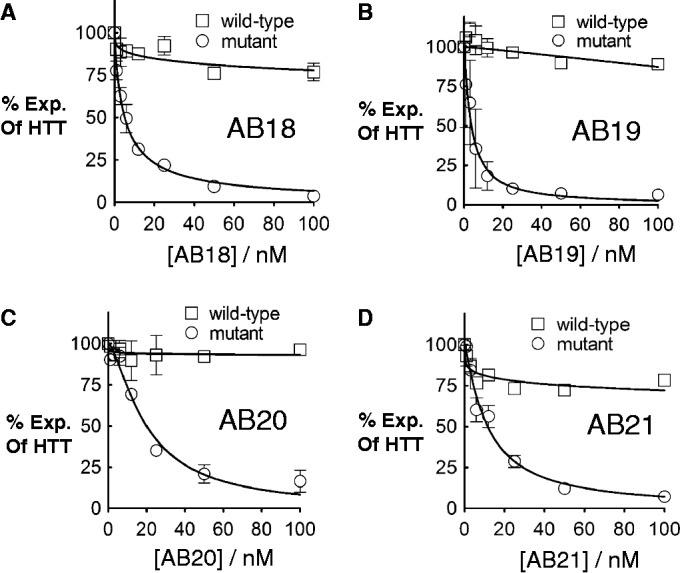
Effects of longer abasic duplex RNAs (22 mers) on expression of HTT. Duplex RNAs were tested in HD patient-derived fibroblast cells (GM04281, CAG 69/17). Averaged dose–response curves of RNA duplexes (**A**) AB18(Y33), (**B**) AB19(Y33), (**C**) AB20(Y16) and (**D**) AB21(Y16) containing three abasic substitutions.

To determine whether an increase in duplex length could compensate for the decrease in potency caused by multiple abasic substitutions, we evaluated duplexes with three abasic substitutions that were 22-base-pairs-long. We found that duplexes AB18 and AB19 were better inhibitors, with IC_50_ values 5.2 nM and 3.8 nM, respectively (Figure 5A and B), than their shorter counterparts. AB20 and AB21 containing three Y16 substitutions had IC_50_ values of 20 nM and 11 nM, respectively (Figure 5C and D and Supplementary Figure S2). The IC_50_ values of the longer duplexes were lower than those of the corresponding 19-base-pair duplexes AB10 and AB15. These suggest that longer duplexes compensate for additional abasic sites.

### Abasic RNAs are allele-selective inhibitors of ATX-3

**Figure 6.**
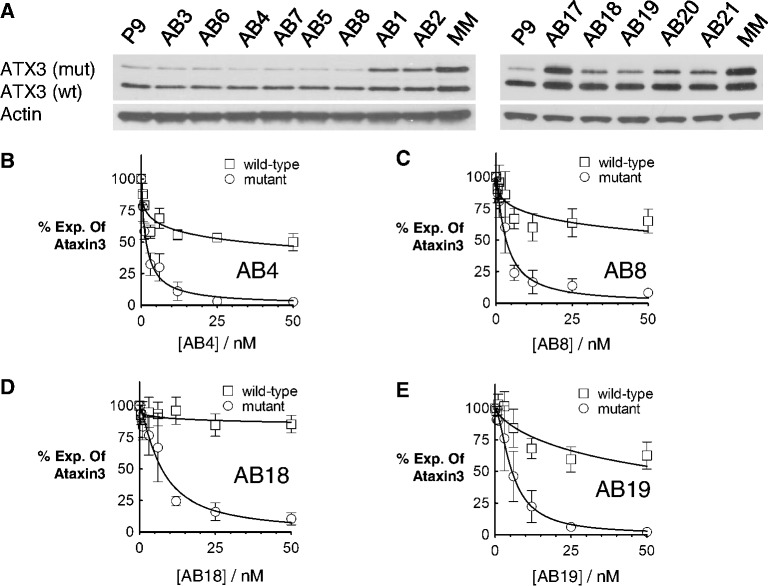
Abasic duplex RNAs selectively inhibit mutant ataxin-3 expression. Duplex RNAs were tested in SCA3 patient-derived fibroblasts (GM06151, CAG 74/24). (**A**) Effects of abasic duplex RNAs on ATX-3 expression when transfected at 25 nM. Averaged western blot dose curves of duplex RNAs (**B**) AB4, (**C**) AB8, (**D**) AB18 and (**E**) AB19 were tested at increased concentrations. Dose curves are data from three independent experiments.

The expanded CAG repeat within the *ATX-3* gene that causes MJD is near the 3′ untranslated region, whereas the expansion in *HTT* that results in HD is near the translation start site. Despite the altered molecular location of the CAG target in the *ATX-3* mRNA, we observed allele-selective inhibition by several different abasic-substituted RNAs (Figure 6A, Table 4). For example, the singly Y34-substituted duplex AB4 had an IC_50_ value of 1.6 nM and a selectivity of 22 fold (Figure 6B). The singly Y33-substituted duplex AB8 had an IC_50_ value of 1.4 nM and a selectivity of 16 fold (Figure 6C). In general, the siRNAs tested against *ATX-3* possessed potencies similar to those for inhibiting HTT expression. As observed for inhibition of HTT, longer duplexes were more tolerant of multiple abasic substitutions and showed improved potencies relative to the standard 19-mer siRNAs (Figure 6D and E and Supplementary Figure S3).

**Table 4.** Inhibition of ataxin-3 expression by duplex RNAs with abasic substitutions

RNA	Mismatch Position	Modification	Mut IC_50_ (nM)	wt IC_50_ (nM)	Selectivity
AB3	G9	Y34	3.7 ± 0.4	38 ± 10	10
AB4	G10	Y34	1.6 ± 0.3	35 ± 14	22
AB5	G11	Y34	2.7 ± 0.5	18 ± 4.6	6
AB6	G9	Y33	4.8 ± 0.4	18 ± 2.4	4
AB7	G10	Y33	1.4 ± 0.2	23 ± 7.2	16
AB8	G11	Y33	3.3 ± 0.6	>100	>30
AB9	G9,10	Y16	3.2 ± 0.4	15 ± 3.2	5
AB20	U8,9,11	Y16	11 ± 2.7	>100	>9
AB21	U8,9,10	Y16	10 ± 2.8	>100	>10
AB18	U8,9,11	Y33	7.2 ± 0.4	>100	>14
AB19	U8,9,10	Y33	5.5 ± 0.9	62 ± 27	11

siRNAs were tested in SCA3 patient fibroblasts GM06151 (CAG,74/24). Selectivity is calculated by comparing the IC_50_ for inhibiting wild-type ATX-3 versus the IC_50_ for inhibiting the mutant allele. The letter “G” or “U” before the number under “Mismatch position” refers to the 3′ terminal base of the antisense strand.

### Melting temperatures, potencies and selectivities

**Figure 7.**
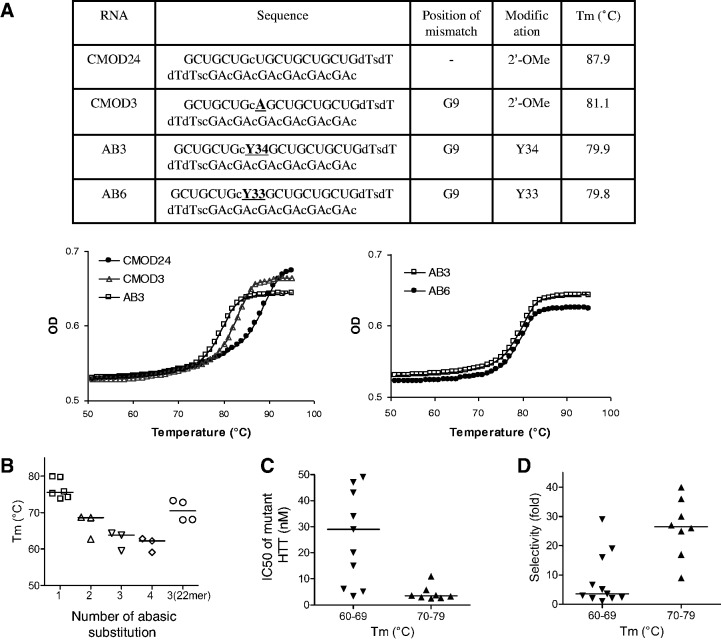
Melting temperature (T_m_) values of analogous chemically modified and abasic RNA duplexes. (**A**) The T_m_ curve on the left compares fully complementary duplex CMOD24, mismatch-containing duplex CMOD3 and analogous abasic duplex AB3. The T_m_ curve on the right compares T_m_ curves for abasic duplexes containing either the Y34 or Y33 substitutions. Correlation of (**B**) abasic substitution number versus T_m_; (**C**) T_m_ versus IC_50_ values for inhibition of mutant HTT; (**D**) T_m_ versus selectivity of abasic duplexes. The letter “G” or “U” before the number under “Mismatch position” refers to the 3′ terminal base of the antisense strand.

We examined the T_m_ values for several abasic-substituted duplexes (Tables 1–3) as a measure of binding affinity for mRNA target. The T_m_s of single abasic Y33- or Y34-substituted RNA duplexes were lower than those of the fully complementary duplex and were similar to a duplex containing a single mismatched base (Figure 7A). T_m_s of the duplexes decreased as the number of abasic substitutions were increased from one to four (Figure 7B). Lengthening the duplex from 19 to 22 base pairs restored T_m_ values for tri-substituted duplexes to those of the parent 19-base-pair duplex with a single substitution. These data indicate that abasic substitutions have greater impact on thermal stability of shorter duplexes and that the reduction in affinity caused by the introduction of abasic moieties can be compensated for by increasing number of base pairs in the duplex.

We also examined the correlation of T_m_s with potency and selectivity. The duplexes with relatively high T_m_ values (70–79°C) were, on average, substantially more potent inhibitors of HTT expression than duplexes with lower values (<69°C) (Figure 7C). Duplexes with high T_m_s also had much higher average allele selectivity (Figure 7D). These data suggest that T_m_ values are an important determinant of both potency and selectivity and that relatively small differences in T_m_ can have large impact on allele-selective inhibition.

### Mechanism of allele-selective inhibition by abasic RNAs

**Figure 8.**
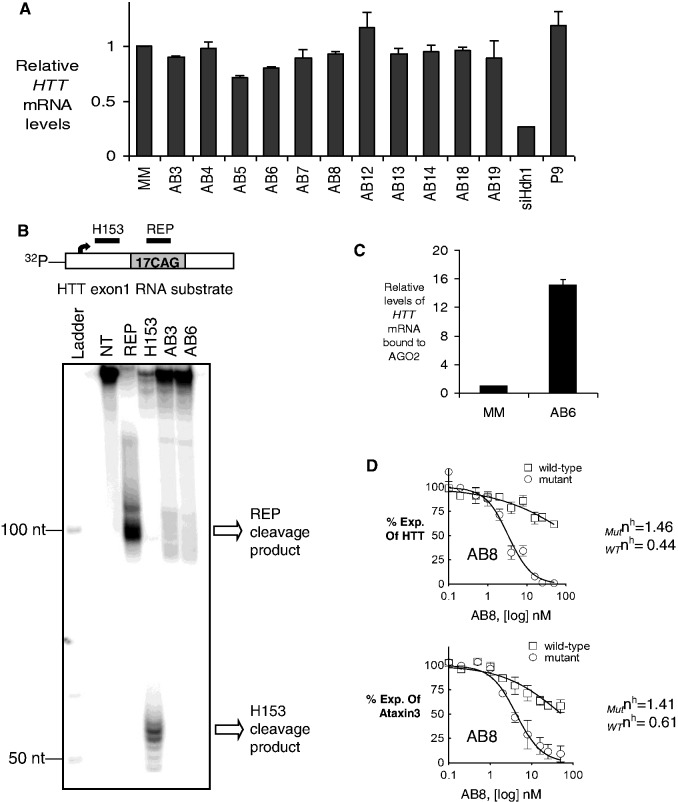
Mechanistic studies of abasic siRNAs. (**A**) *HTT* mRNA levels after treating with 25 nM of abasic duplex RNAs in HD patient fibroblasts (GM04281, CAG 69/17). siHdh1 is a positive control siRNA targeting *HTT* mRNA at a sequence outside the trinucleotide repeat region. (**B**) *In vitro* cleavage assay using RNA antisense strands and recombinant human AGO2 protein. Ladder: radiolabeled 10-nt DNA markers; NT: no treatment; REP: fully complementary anti-CAG siRNA; H153: fully complementary siRNA targeting *HTT* upstream region of CAG repeat; AB3 and AB6 are abasic-substituted siRNAs. (**C**) RNA immunoprecipitation (RIP) using an anti-AGO2 antibody reveals association of AGO2/siRNA complexes with *HTT* mRNA (GM04281). (**D**) Abasic siRNA AB8 selectively inhibits mutant HTT or ataxin-3 expression in a cooperative manner.

RNAi with fully complementary duplexes generally leads to AGO2-mediated cleavage of the target mRNA and reduced mRNA levels (36–38). To determine how abasic substitutions might affect this fundamental aspect of RNAi, we examined levels of *HTT* mRNA after transfection of abasic duplexes into GM04281 fibroblast cells. We observed little or no alteration of mRNA levels (Figure 8A) when cells were treated with concentrations of siRNA that reduced HTT protein levels.

We also assayed cleavage of a synthetic radiolabeled substrate containing the CAG repeat. The antisense strands of active duplexes were individually incubated with AGO2 protein and then the loaded AGO2 was mixed with the radiolabeled CAG repeat. Target cleavage was observed when the duplex RNA was fully complementary, but not when the antisense strand of abasic duplex AB3 or AB6 was used (Figure 8B).

RNA immunoprecipitation (RIP) with an anti-AGO2 antibody further confirmed the involvement of RNAi machinery in reduction in HTT levels in cells treated with duplexes containing abasic modifications. The RIP assay demonstrated that addition of duplex AB6 caused recruitment of AGO2 to *HTT* mRNA (Figure 8C). Taken together, our data indicate that a guide strand with one or more abasic substitutions close to or around the cleavage site does recruit AGO2 to the target mRNA, but the endonuclease activity of AGO2 is not activated.

We had previously observed that unmodified duplex RNAs (14) act cooperatively to inhibit HTT expression. To test whether abasic RNAs would also show cooperative inhibition, we examined allele-selective inhibition of HTT and ATX-3 protein production. Duplex AB8 was transfected into GM04281 HD or GM06151 fibroblasts and protein expression was monitored over a broad range of concentrations. We fitted the data to the Hill equation (39) and obtained Hill coefficients of 1.46 and 1.41 for inhibition of mutant HTT and ATX-3 expression, respectively. The Hill coefficients for inhibition of wild-type HTT and wild-type ATX-3 were lower, 0.44 and 0.61, respectively (Figure 8D and Supplementary Figure S4). These data are consistent with cooperative inhibition of mutant relative to wild-type protein expression.

## DISCUSSION

Nucleic acids are valuable tools for research and promising therapeutic agents. Much of their promise relative to small-molecule drugs is due to the inherent highly selective recognition of RNA targets through Watson–Crick base-pairing. The recent Food and Drug Administration approval of the single-stranded antisense compound Kynamro (40) for treating familial hypercholesterolemia highlights the potential therapeutic value of nucleic acids and the feasibility of their systemic application. Continued progress, however, will confront substantial challenges, and chemical modifications will play a major role in optimizing nucleic acids for therapy (1).

Abasic substitutions provide a relatively unexplored strategy for modulating chemical, thermodynamic and protein-recognition properties of oligonucleotides (2–10). Our goal here was to test how abasic RNA compared with mismatched RNA for allele-selective inhibiting expression of *HTT* and *ATX-3*. Mutant HTT and ATX-3 protein exert their deleterious effects in the central nervous system. Treatments for HD or MJD are urgently needed, but the requirement for action in the brain complicates delivery. As a result, it is important to establish the full range of chemical options that can achieve potent and selective inhibition so that optimally effective inhibitors can be identified.

Mismatches and abasic substitutions are structurally different and confer different physical properties on duplexes that contain them. Despite their differences, we find that duplexes with one or two abasic substitutions inhibit HTT or ATX-3 expression with selectivity similar to analogous mismatch-containing duplexes. Duplexes with three or four abasic substitutions in the central region of the guide strand showed a clear reduction in potency with 19-mer base-pair duplex relative to the mismatched duplexes, but this could be compensated for when the length of the parent duplex was increased to 22 base pairs.

It has been shown previously that the introduction of mismatches at central positions within an RNA duplex disrupts cleavage of target RNA by AGO2 (16). Here we show that central abasic substitutions also block the slicer function of AGO2 (Figure 8B). These data add abasic substitutions to the strategies for manipulating AGO2 activity and provide more data on the limits on how AGO2 tolerates imperfect recognition of substrate.

Three different abasic substitutions were used in these studies (Figure 1C), and their similarities are much more obvious than any differences. Duplexes with the Y16, Y33 and Y34 modifications possessed similar potencies and allele selectivities. For example, we can directly compare Y34 duplexes AB3 to AB5 with Y33 duplexes AB6 to AB8 because both sets have substitutions at positions 9, 10 or 11. All six duplexes had potencies with a narrow range of 2.5 to 5.8 nM. For the Y34 duplexes, allele selectivities ranged from 25 to >40 fold, similar to selectivities for the Y33 duplexes, >17 to >30 fold. The similarity of these values suggests that the structural consequences of abasic recognition on AGO2 action are similar regardless of which abasic substitution is used. Based on the limited data set presented here, all three abasic substitutions appear to be effective and our work reveals no obvious preference for one over another.

Argonaute protein adopts an RNase H-like fold with three catalytic residues lining the active site (41). Based on crystal data for argonaute in complex with both guide and fully matched target strand, it can be seen that an exact coordination geometry of the 3′-O leaving group of the scissile phosphate to a divalent cation is required for in line attack of a water molecule to occur. From these crystal data, it can also be seen that perfect base-pairing of the guide strand to the target is essential for this geometry to be assumed. In particular, correct base-pairing at positions 9 and 10 is necessary for proper positioning of catalytic residues.

We find it a reasonable assumption that should abasic sites be positioned opposite to the scissile phosphate, an increase in flexibility for the target strand would also result. This increase in flexibility would perhaps result in only transient presentation of the geometric constraints necessary for hydrolysis. This additional flexibility would decrease catalysis and RNA cleavage. Multiple abasic substitutions further increase flexibility, but the increase is so large that it also begins to affect the affinity of base-pairing and causes a decrease in the potency of inhibition.

Abasic duplexes will provide a novel starting point for allele-selective inhibition of the expression of the genes that cause CAG-repeat pathologies. In combination with other chemical modifications or strategically placed mismatches, the inclusion of abasic sites might lead to development of duplexes with optimized potency and selectivity. It is also possible that abasic substitutions may have subtle influence on biodistribution, and this impact may be favorable. Our data demonstrate that abasic substitutions are compatible with the development of potent silencing agents, are predictable modulators of the physical properties of parent duplexes and merit wider attention than they have received to date.

## SUPPLEMENTARY DATA

Supplementary Data are available at NAR Online, including [42].

Supplementary Data
