# Supplementary material for: RNA duplexes with abasic substitutions are potent and allele-selective inhibitors of huntingtin and ataxin-3 expression
Source: Nucleic Acids Res. 2013 Jul 24;41(18):8788–801. doi: 10.1093/nar/gkt594 (PMC3794577; doi:10.1093/nar/gkt594)
Supplement: Supplementary Data [file supp_41_18_8788__index.html]

RNA duplexes with abasic substitutions are potent and allele-selective inhibitors of huntingtin and ataxin-3 expression — RNA duplexes with abasic substitutions are potent and allele-selective inhibitors of huntingtin and ataxin-3 expression — Supplementary Data 

# RNA duplexes with abasic substitutions are potent and allele-selective inhibitors of huntingtin and ataxin-3 expression

## 

files

**Files in this Data Supplement:**

- Supplementary Data - pdf file
