# Supplementary material for: RNA duplexes with abasic substitutions are potent and allele-selective inhibitors of huntingtin and ataxin-3 expression
Source: Nucleic Acids Res. 2013 Jul 24;41(18):8788–801. doi: 10.1093/nar/gkt594 (PMC3794577; doi:10.1093/nar/gkt594)

Supplementary Method. Characterization of abasic phosphoramidite monomers

Synthesis of compound **2**: 4-(Dimethylamino)pyridine (DMAP, 2.31 g, 18.9 mmol) and 4,4′-dimethoxytriphenylmethyl chloride (DMTr-Cl, 64 g, 0.189 mol) were added to a stirring solution of (2*R*,3*S*)-2-(hydroxymethyl)tetrahydro-2H-pyran-3-ol[1] (1,5-anhydro-2,3-dideoxy-D-erythro-hexitol or compound **1**, 25 g, 0.189 mol) in anhydrous pyridine (200 mL) under argon atmosphere at ambient temperature. The stirring was continued for 2 h. Solvent was removed *in vacuo* at ambient temperature, and then the residue was co-evaporated with toluene to remove residual pyridine. The residue was dissolved in ethyl acetate (500 mL) washed with saturated bicarbonate solution followed by water, dried over anhydrous sodium sulfate, and solvents and volatiles removed under reduced pressure. The crude product was purified by silica gel (deactivated with 2% triethylamine) chromatography using 10-50% ethyl acetate in hexane as eluent to obtain the compound **2** (44 g, 53%) as an off-white solid. ^{1}H NMR (400 MHz, $CDCl_3$): δ1.37-1.47 (m, 1H), 1.62-1.64 (m, 2H), 2.08-2.22 (m, 1H), 3.21-3.22 (m, 2H), 3.26-3.35 (m, 2H), 3.45- 3.55 (m, 2H), 3.78 (s, 6h), 3.85-3.87 (m, 1H), 6.82- 6.84 (d, *J* =8.8 Hz, 4H), 7.19-7.23 (t, 1H), 7.26-7.32(m, 6H), 7.40- 7.43 (m, 2H). ^{13}C NMR (100 MHz, $CDCl_3$): 24.83, 31.40, 55.03, 66.07, 67.50, 70.00, 79.20, 86.89, 113.11, 126.77, 127.47, 129.82, 135.23, 135.45, 144.23, and 158.41. Mass calc. for $C_{27}H_{30}O_5$ 434.21; found 457.2 (M+23).

Synthesis of phosphoramidite **3**: To a solution of compound **2** (5.00 g, 11.50 mmol) in anhydrous dichloromethane (50 mL) was added DIEA (4.00 mL, 2 eq.) followed by 2-cyanoethyl *N*,*N*-diisopropylchlorophosphoramidite (3.26 g, 1.2 eq) under argon at ambient temperature. The mixture was stirred for 30 min, diluted with DCM (100 mL), and washed with water and bicarbonate solution. The organic layer was dried over anhydrous sodium sulfate and evaporated *in vacuo*, and the crude product was purified by flash silica gel column chromatography using 10-30% EtOAc in hexane as eluent to obtain the phosphoramidite **3** as white fluffy solid (6.86 g, 94%). ^{1}H NMR (400 MHz, DMSO-d_6): δ 7.47 – 7.31 (m, 2H), 7.31 – 7.12 (m, 7H), 6.84 (dd, *J* = 8.6, 7.0 Hz, 4H), 4.07 – 3.93 (m, 1H), 3.92 – 3.79 (m, 1H), 3.70 (d, *J* = 2.2 Hz, 6H), 3.66 – 3.43 (m, 2H), 3.42 – 3.20 (m, 7H), 2.92 (m, 1H), 2.68 (t, *J* = 5.8 Hz, 1H), 2.14 (d, *J* = 10.1 Hz, 1H), 1.68 – 1.30 (m, 3H), 1.19 – 0.88 (m, 10H), 0.78 (d, *J* = 6.7 Hz, 3H). ^{31}P (160 MHz, DMSO-d_6): δ 151.19, 149.89.

Synthesis of compound **5**: Commercially available 1-deoxy-D-ribofuranose (**4,** 12.66 g, 94.44 mmol) was dissolved in anhydrous pyridine (200 ml). The reaction mixture was cooled in an ice-water bath under argon. 1,3-Dichloro-1,1,3,3-tetraisopropyldisiloxane (32.21 ml, 100.7 mmol) was added drop wise to the solution with stirring. Stirring was continued overnight. The mixture was cooled over an ice bath, and 10 mL of water was added. Volatiles were removed *in vacuo* and the residue was extracted into ethyl acetate, and washed with water, with saturated sodium bicarbonate solution, and with brine. The crude product was purified by flash silica gel column chromatography using 5-30% ethyl acetate in hexanes as eluent to obtain compound **5** as a white solid (27.20 g, 76%). ^{1}H NMR (400 MHz, DMSO-d$_6$): δ 4.68 (d, *J* = 3.5 Hz, 1H), 4.06 (m, 2H), 3.94 (dd, *J* = 9.7, 3.9 Hz, 1H), 3.85 (t, *J* = 3.5 Hz, 2H), 3.65 (dt, *J* = 7.4, 3.5 Hz, 1H), 3.54 (dd, *J* = 9.7, 0.9 Hz, 1H), 1.09 – 0.88 (m, 28H). ^{13}C NMR (101 MHz, CDCl$_3$): δ 75.50, 68.31, 67.79, 65.74, 57.32, 12.19, 12.07, 12.05, 11.94, 11.78, 11.71, 8.11, 7.89, 7.57, 7.40. Mass calc. for $C_{17}H_{36}O_5Si_2$ 376.21; found 377.20 (M+H).

Synthesis of compound **6**: Compound **5** (2.23 g, 5.92 mmol) was added to anhydrous DMF and THF (2:1, 30 ml) and cooled the mixture to -50 °C under argon. NaH (0.284 g, 60 wt% in mineral oil, 1.2 eq) was added slowly; methyl iodide (0.50 mL, 7.70 mmol) was then added to the reaction mixture. During addition the reaction temperature was maintained around -50 °C. The reaction mixture was slowly warmed to about -20 °C and stirred for 20 h. The reaction mixture was cooled to -50 °C and was quenched with cold ammonium chloride solution and diluted with ethyl acetate (100 mL). The organic layer was washed with water and brine. Solvents were removed *in vacuo*, and the crude product was purified by flash silica gel column chromatography using 5-30% ethyl acetate in hexanes as eluent to obtain compound **6** as colorless liquid (1.30 g, 55%). ^{1}H NMR (400 MHz, DMSO-d$_6$): δ 4.15 (dd, *J* = 8.9, 4.5 Hz, 1H), 3.97 (dd, *J* = 10.2, 4.4 Hz, 1H), 3.82 (m, 3H), 3.68 – 3.58 (m, 2H), 3.44 (s, 3H), 1.13 – 0.89 (m, 28H). ^{13}C NMR (101 MHz, CDCl$_3$) δ 75.50, 68.31, 67.79, 65.74, 57.32, 12.19, 12.07, 12.05, 11.94, 11.78, 11.71, 8.11, 7.89, 7.57, 7.40. Mass calc. for $C_{18}H_{38}O_5Si_2$ 390.22; found 391.2 (M+H).

Synthesis of **7**: Compound **6** (4.47 g, 11.45 mmol) was dissolved in a mixture of acetonitrile and dichloromethane (3:1, 40 mL) and transferred to a Teflon vessel. A solution of hydrogen fluoride in triethylamine (10 mL) was added, and the mixture was stirred overnight. After completion of the reaction (~20 h), sodium bicarbonate solution (100 mL) and dichloromethane (100 mL) were added. The resulting suspension was stirred for 20 min and was then filtered. The residue was

washed with dichloromethane (50 mL). The combined filtrates were evaporated under reduced pressure, and the residue was purified by flash silica gel column using 0-20% MeOH in dichloromethane as eluent to obtain compound **7** as a colorless liquid (1.44 g, 85%). ^{1}H NMR (400 MHz, DMSO-d_6): δ 4.67 (d, J = 6.5 Hz, 1H), 4.57 (t, J = 5.7 Hz, 1H), 3.91 (dd, J = 11.5, 5.9 Hz, 1H), 3.84 (dd, J = 9.0, 5.0 Hz, 1H), 3.69 (dd, J = 9.4, 4.7 Hz, 1H), 3.60 (m, 2H), 3.55 – 3.43 (m, 1H), 3.41 – 3.29 (m, 4H). ^{13}C NMR (126 MHz, DMSO-d_6): δ 83.66, 80.29, 70.84, 69.09, 61.62, 57.21. Mass calc. for $C_6H_{12}O_4$ 148.07; found 149.1 (M+H).

<u>Synthesis of **8**</u>: DMAP (200 mg) and DMTr-Cl (3.55 g, 10.18 mmol) were added to a solution of **7** (1.43 g, 9.70 mmol) in anhydrous pyridine (50 mL) at ambient temperature and stirred overnight. Solvents were removed, and the residue was dissolved in ethyl acetate, then washed with water and brine. The crude product was purified by flash silica gel column chromatography using 10-70 % ethyl acetate in hexanes as eluent to obtain the compound **8** (3.53 g, 81%) as a pale yellow solid. ^{1}H NMR (400 MHz, DMSO-d_6): δ 7.41 (d, J = 7.6 Hz, 2H), 7.36 – 7.16 (m, 7H), 6.89 (d, J = 8.8 Hz, 4H), 4.81 (d, J = 7.0 Hz, 1H), 3.97 – 3.86 (m, 2H), 3.84 – 3.70 (m, 3H), 3.67(s, 6H) 3.34 (s, 3H), 3.11 (dd, J = 9.9, 2.6 Hz, 1H), 2.96 (dd, J = 9.9, 5.6 Hz, 1H). ^{13}C NMR (126 MHz, DMSO-d_6): δ 157.97, 144.98, 135.70, 135.67, 129.66, 127.72, 127.68, 126.54, 113.09, 85.14, 81.32, 80.08, 71.66, 69.39, 64.26, 57.17, 54.96. MS calc. for $C_{27}H_{30}O_6$, 450.20; found 451.2 (M+H).

<u>Synthesis of **9**</u>: DIEA (2.60 mL, 15 mmol) and 2-cyanoethyl *N,N*-diisopropylchlorophosphoramidite (1.90 g, 7.92 mmol) were added to a solution of **8** (3.40 g, 7.55 mmol) in dichloromethane (50 mL) at ambient temperature, and the reaction was stirred for 30 min. The reaction mixture was washed with saturated sodium bicarbonate solution and water. Solvents were removed *in vacuo*, and the crude product was purified by flash silica gel column chromatography using 10-60% ethyl acetate in hexanes as eluents to obtain phosphoramidite **9** (4.30 g, 88%) as a colorless gummy liquid. ^{1}H NMR (400 MHz, DMSO-d_6): δ 7.40 (dd, *J* = 7.2, 4.5 Hz, 2H), 7.35 – 7.15 (m, 7H), 6.88 (dd, *J* = 8.8, 5.1 Hz, 4H), 4.27 – 3.82 (m, 3H), 3.81 – 3.66 (m, 7H), 3.50 (m, 3H), 3.39 – 3.28 (m, 3H), 3.20 (m, 1H), 2.96(m 1H), 2.76 (t, *J* = 5.9 Hz, 1H), 2.56 (t, *J* = 5.9 Hz, 1H), 1.11 (dd, *J* = 21.1, 11.9 Hz, 11H), 0.92 (d, *J* = 6.7 Hz, 3H). ^{31}P NMR (162 MHz, DMSO-d_6): δ 153.52, 153.39.

1.Nicolaou, K. C.; Hwang, C. K.; Marron, B. E.; DeFrees, S. A.; Couladouros, E. A.; Abe, Y.; Carroll, P. J.; Snyder, J. P. *J. Am. Chem. Soc.* **1990**, *112*, 3040.

Supplementary Figure 1. Effects of siRNAs with multiple abasic modifications on expression of HTT. Duplex RNAs were tested in HD patient-derived fibroblast cells (GM04281, CAG 69/17). (**A**) Averaged protein levels of HTT expression after transfection of duplex RNAs at 25 nM. (**B**) Representative western blot images of duplex RNA/AB9 and AB12.

A

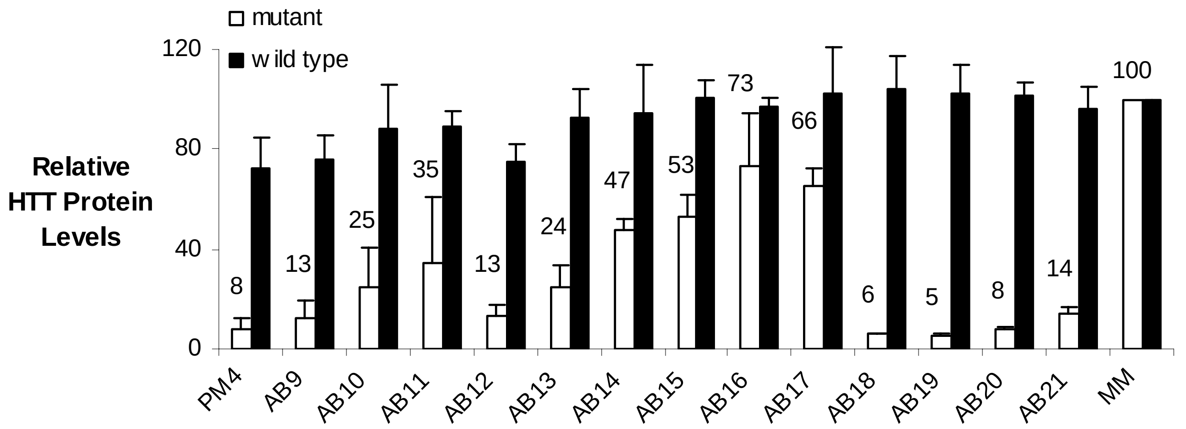

B

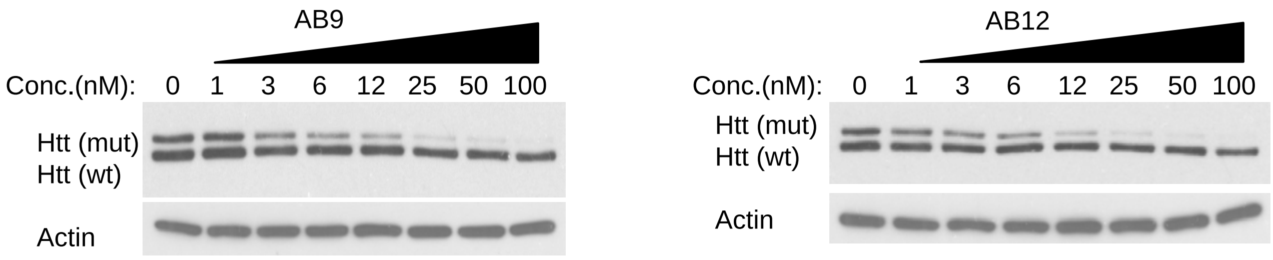

Supplementary Figure 2. Effects of longer abasic duplex RNAs (22mers) on expression of HTT expression. Duplex RNAs were transfected into HD patient-derived fibroblast cells (GM04281, CAG 69/17). Representative western blot image of RNA duplexes/AB18, AB19, AB20, and AB21.

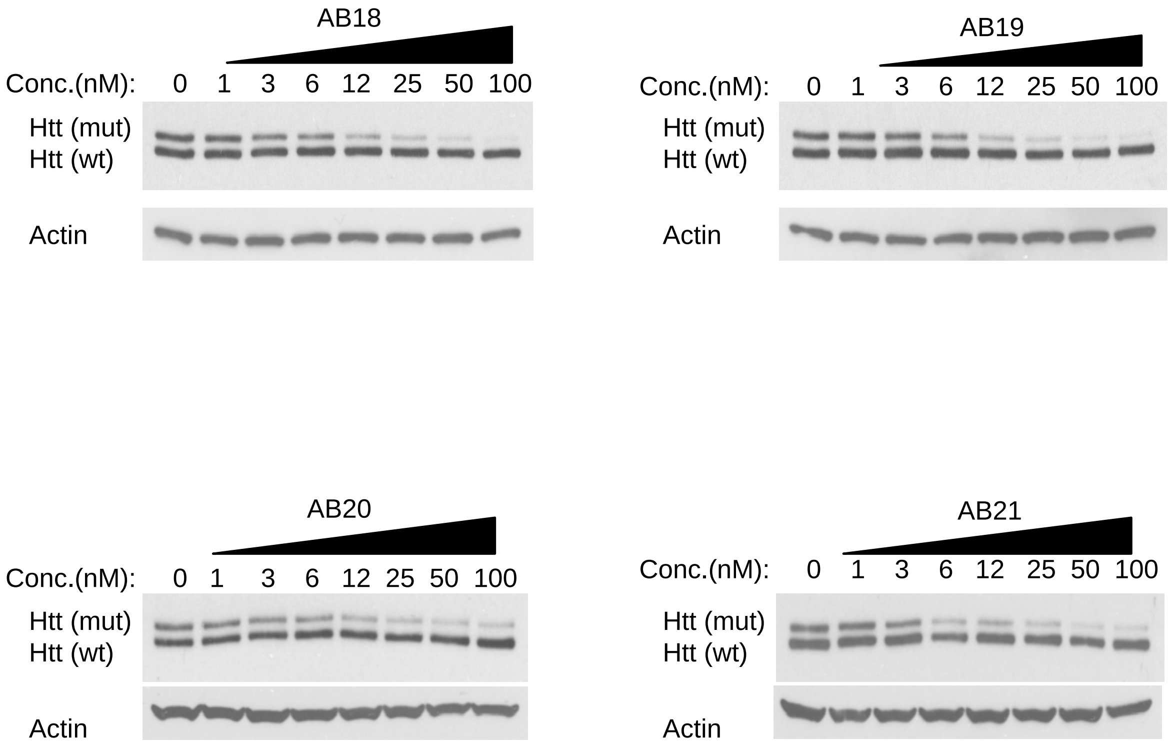

Supplementary Figure 3. Abasic duplex RNAs selectively inhibit expression of mutant ataxin-3. Duplex RNAs were tested in SCA3 patient-derived fibroblasts (GM06151, CAG 74/24). Representative western blot images of duplex RNA/AB4, AB8, AB18, and AB19.

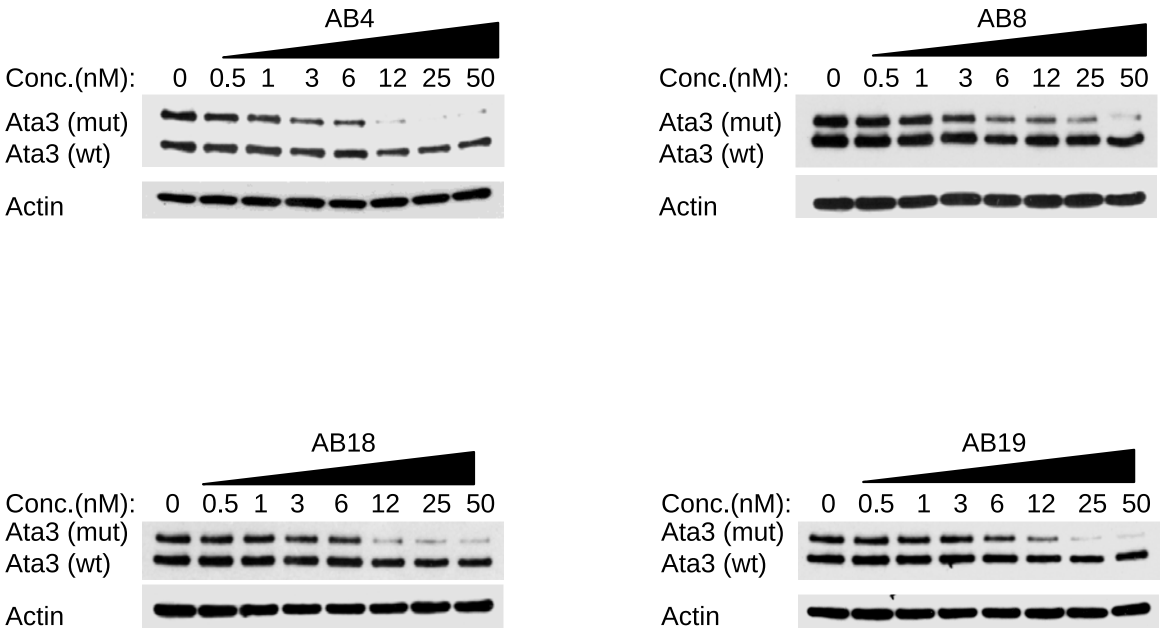

Supplementary Figure 4. Abasic siRNAs selectively inhibit mutant HTT or ataxin-3 expressions in a cooperative manner. Representative western blot images of abasic siRNA/AB8 tested in (**A**) HTT fibroblasts (GM04281, CAG 69/17) or (**B**) SCA3 fibroblasts (GM06151, CAG 74/24) at increased concentrations.

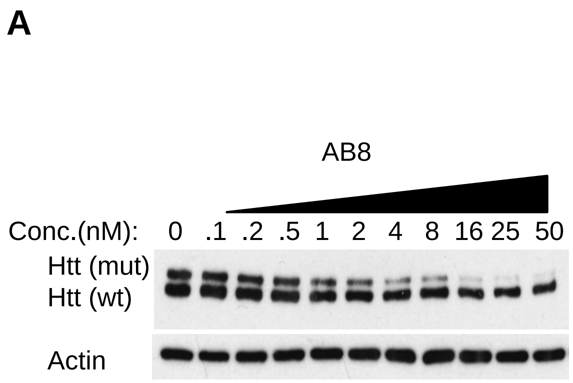

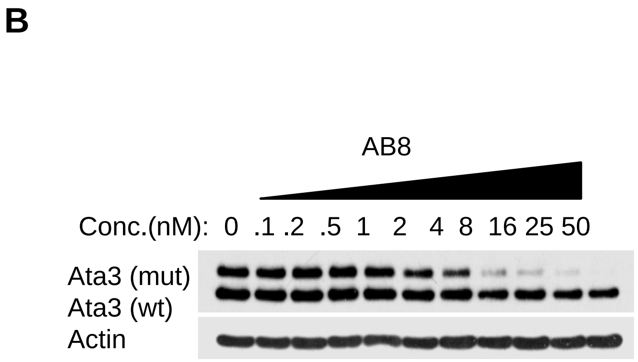

Supplement: Supplementary Data [file supp_gkt594_nar-01279-h-2013-File004.pdf]
